# Pulmonary hypertension in dialysis patients: clinical insights from tricuspid regurgitation velocity (TRV)–based echocardiographic criteria

**DOI:** 10.1080/0886022X.2026.2639889

**Published:** 2026-03-18

**Authors:** Sukrisd Koowattanatianchai, Patchara Kochaiyapatana, Metus Kanokwatanakul, Kiraphol Kaladee, Chatchai Kreepala

**Affiliations:** a Division of Cardiology, Department of Internal Medicine, Burapha University, Chonburi, Thailand; b Department of Internal Medicine, Burapha University, Chonburi, Thailand; c School of Health Science, Sukhothai Thammathirat Open University, Nonthaburi, Thailand; d Nephrology Unit, School of Internal Medicine, Institute of Medicine, Suranaree University of Technology, Nakhon Ratchasima, Thailand

**Keywords:** Pulmonary hypertension, end-stage renal disease, Tricuspid regurgitation velocity, echocardiography, dialysis

## Abstract

Pulmonary hypertension (PH) is a frequent yet underrecognized complication in end-stage renal disease on dialysis. Earlier echocardiographic definitions based on pulmonary artery systolic pressure (PASP) >35 mmHg derived from right atrial pressure estimation were prone to inaccuracy and volume overload bias. In 2022, the European Society of Cardiology/European Respiratory Society introduced tricuspid regurgitation velocity (TRV)–based criteria incorporating structural and functional markers to improve diagnostic specificity.

We aimed to determine the prevalence of PH in dialysis patients using TRV-based echocardiographic criteria and to identify independent predictors. This retrospective cross-sectional study was conducted at Burapha University Hospital, Chonburi Province, Thailand, from October 1, 2023, to October 30, 2024, and included 117 dialysis patients who underwent echocardiography. PH was defined as TRV >2.8 m/s plus ≥1 additional echocardiographic sign. The prevalence of PH was 43.6% (51/117). Compared with non-PH patients, those with PH had greater left ventricular diastolic dimensions, higher left atrial volume index, more diastolic dysfunction, and more frequent mitral regurgitation (MR). In multivariable analysis, longer dialysis vintage (adjusted relative risk [ARR] 1.50 per year, *p* = 0.009), larger LV diastolic dimension (ARR 1.06 per mmHg, *p* = 0.036), and the presence of MR (ARR 2.00, *p* = 0.020) were independent predictors.

PH was common in dialysis patients even when applying stricter TRV-based criteria that reduce misclassification related to volume status. The observed associations with dialysis vintage, left ventricular remodeling, and MR suggest a predominance of post-capillary mechanisms and support a heart–kidney–lung interaction.

## GRAPHICAL ABSTRACT

**Figure:**
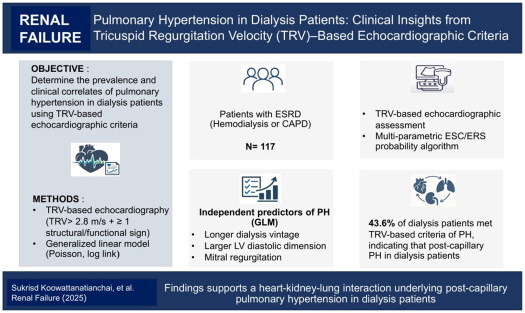

## Introduction

Pulmonary hypertension (PH) is a frequent and clinically significant complication in chronic kidney disease (CKD), particularly among patients with end-stage renal disease (ESRD) receiving dialysis. Reported prevalence ranges from approximately 17–56% in hemodialysis (HD) patients by echocardiography [1,2], and up to 77% by right heart catheterization [3]. It is associated with increased cardiovascular morbidity and mortality, yet there are currently no treatment guidelines specifically targeting PH in ESRD [4]. Accurate identification is also important from a healthcare resource perspective, as misclassification can lead to unnecessary investigations and treatments, increasing economic burden similar to inefficiencies in inpatient care [5]. Notably, experience from other areas of nephrology suggests that the introduction of more refined diagnostic frameworks does not necessarily lead to a marked reduction in disease prevalence estimates [6,7]. Similarly, studies applying updated echocardiographic criteria in non-dialysis populations have reported persistently high rates of echocardiographic PH, despite stricter definitions.

Previous echocardiography-based studies often defined PH as pulmonary artery systolic pressure (PASP) >35 mmHg, which requires estimation of right atrial pressure (RAP) from inferior vena cava parameters [8–11]. This method is prone to inaccuracies due to interobserver variability and error amplification when RAP is incorporated into derived variables such as PASP. Reliance on tricuspid regurgitation velocity (TRV) alone is also limited by its sensitivity to volume overload—a particularly important confounder in ESRD. In HD patients, echocardiography may be performed either before or after dialysis, often without standardization. If performed before HD, especially after multiple days without treatment, significant volume overload can transiently increase TRV and produce false-positive diagnoses. In contrast, continuous ambulatory peritoneal dialysis (CAPD) maintains more stable volume status, potentially explaining the lower observed prevalence of PH. Similar variability has been observed in other dialysis-related hemodynamic assessments [12].

In 2022, the European Society of Cardiology (ESC) and European Respiratory Society (ERS) introduced TRV-based echocardiographic criteria for assessing the probability of PH [13]. Unlike the older PASP >35 mmHg definition, which relies heavily on estimated RAP, the TRV-based approach requires both elevated TRV (>2.8 m/s) and additional echocardiographic features from at least one of three domains: right ventricular morphology and function, pulmonary artery parameters, or right atrial and inferior vena cava dimensions. This combined functional–structural requirement addresses key shortcomings of the old method by reducing overdiagnosis from transient hemodynamic changes, particularly volume overload in HD patients, and improving specificity for true pulmonary vascular pathology [13]. Similar multi-parametric approaches have improved diagnostic accuracy in other renal and cardiovascular conditions, as seen in biomarker-driven studies for early kidney disease detection and transplant pathology [14,15]. Importantly, this guideline was developed with recognition that standardized pre- or post-dialysis timing is rarely feasible in ESRD practice. By reducing reliance on preload-sensitive parameters such as RAP and IVC collapsibility, it offers a more stable diagnostic framework across fluctuating volume states, which is particularly relevant in HD patients. To date, no study has systematically applied the full 2022 ESC/ERS echocardiographic probability algorithm specifically in patients receiving chronic dialysis. This study addresses this gap by evaluating PH prevalence and associated factors in a dialysis-only cohort using the updated multi-parametric criteria.

Although PH is a recognized complication in ESRD and has been linked to increased cardiovascular events and mortality [16], its reported prevalence in dialysis populations remains inconsistent. Variations in diagnostic criteria and timing of echocardiography relative to dialysis sessions ^10^ contribute to this discrepancy. These limitations also hinder accurate identification of underlying risk factors, as misclassification can weaken or obscure true associations.

To address these gaps, we conducted a retrospective cross-sectional study in ESRD patients on HD and CAPD to determine the prevalence of PH using the TRV-based echocardiographic definition from the 2022 ESC/ERS guidelines and to identify clinical risk factors from routinely collected clinical and laboratory data.

## Methods

### Participants

This retrospective cross-sectional study was conducted at Burapha University Hospital, Chonburi Province, Thailand, from October 1, 2023, to October 30, 2024.

Eligible participants were adults aged >18 years with a confirmed diagnosis of ESRD who were receiving renal replacement therapy with either HD or peritoneal dialysis (PD), and who had complete clinical and echocardiographic data available.

Patients were excluded if they had any of the following:

1. Congenital heart disease;
2. Significant valvular heart disease, defined as moderate-to-severe primary valvular disease of structural or rheumatic origin (mild functional valvular regurgitation was not an exclusion criterion);
3. Primary idiopathic PH;
4. Chronic obstructive pulmonary disease;
5. Acute or chronic pulmonary embolism; or
6. HIV infection.

The study protocol was reviewed and approved by the Burapha University Institutional Review Board (approval number: HS113/2565) and conducted in accordance with the Declaration of Helsinki. Given the retrospective design, the requirement for written informed consent was waived by the ethics committee.

### Clinical and laboratory data collection

Demographic, clinical, and laboratory data were retrieved from medical records. Variables included age, sex, body mass index (BMI), comorbidities, etiology of CKD, dialysis vintage, and current medications. Dialysis modality was recorded as either HD *via* arteriovenous fistula or arteriovenous bridge graft, or CAPD, consistent with the study’s inclusion criteria. Patients dialyzed through permanent catheters or double-lumen catheters were excluded. Atrial fibrillation (AF) was identified from electrocardiographic (ECG) findings diagnosed and documented by cardiologists in the patients’ records, with both chronic persistent AF and paroxysmal AF episodes included in the analysis. Laboratory parameters comprised blood urea nitrogen (BUN), serum creatinine (Cr), hemoglobin, calcium, phosphorus, albumin, and parathyroid hormone (PTH) levels. Blood samples in HD patients were collected midweek, representing the longest interdialytic interval, whereas samples in CAPD patients were obtained in the morning before initiating treatment on a convenient day.

### Echocardiography

Transthoracic echocardiographic data were reviewed by two experienced cardiologists (SK and PK) who were blinded to the patients’ clinical information. Discrepancies in interpretation or measurement were resolved by consensus.

PH was diagnosed when TRV exceeded 2.8 m/s and at least one additional echocardiographic feature suggestive of PH was present. Additional features were categorized into three domains [13]:

1. Ventricular findings: right ventricle/left ventricle (RV/LV) basal diameter or area ratio >1.0; interventricular septal flattening (left ventricular eccentricity index >1.1 in systole and/or diastole); TAPSE/systolic pulmonary artery pressure (sPAP) ratio <0.55 mm/mmHg.
2. Pulmonary artery findings: right ventricular outflow tract acceleration time (RVOT AT) <105 ms and/or mid-systolic notching; early diastolic pulmonary regurgitation velocity >2.2 m/s; pulmonary artery (PA) diameter > aortic root (AR) diameter and >25 mm.
3. Inferior vena cava (IVC) and right atrium (RA) findings: IVC diameter >21 mm with reduced inspiratory collapse (<50% with a sniff or <20% with quiet inspiration); RA area at end-systole >18 cm^2^.

Importantly, patients in whom TRV could not be measured were not excluded from the study. In these cases, the absence of a measurable TRV reflected the absence of a tricuspid regurgitation jet, rather than suboptimal image quality. To avoid misclassification and potential underestimation of PH prevalence, mean pulmonary arterial pressure (mPAP) was estimated using a Doppler-derived method only when TRV was unavailable, while the TRV-based 2022 ESC/ERS algorithm remained the primary framework for PH classification.

Systolic pulmonary artery pressure was calculated as:

$$\text{sPAP}=\text{ TR pressure gradient }+\text{ estimated right atrial pressure }\left(\text{RAP}\right)$$

RAP was estimated from IVC diameter and degree of inspiratory collapse, categorized as 3, 8, or 15 mmHg for normal, moderate, or elevated pressure, respectively.

If TRV could not be measured, mean pulmonary artery pressure (mPAP) was calculated using Abbas’s formula [17]:

$$\text{mPAP}=4\times {\left(\text{PRV}\right)}^{2}+\text{RAP}$$

At rest, PH was defined as an estimated mPAP >20 mmHg.

### Dialysis timing consideration

Echocardiography was performed as part of routine clinical practice without standardized pre- or post-dialysis scheduling. Previous cardiology evidence has shown that intravascular volume status does not materially affect TRV, as TRV elevation in PH is driven predominantly by increased right-sided pressures rather than preload. Consistent with these data, the 2022 ESC/ERS guideline does not require timing standardization for TRV-based assessment. Therefore, varying interdialytic intervals in HD patients were considered acceptable and aligned with the intention of the guideline’s multiparametric TRV-based framework.

For quality control, TRV measurements were included only when the continuous-wave Doppler envelope demonstrated a complete, well-defined, and dense signal with clear peak velocity. Incomplete, low-quality, or poorly aligned envelopes were not accepted. All TRV measurements were independently reviewed by two cardiologists, with discrepancies resolved by consensus.

### Statistical analysis

Data analysis was conducted using Stata version 17.0 (StataCorp, College Station, TX, USA). Categorical variables were summarized as frequencies and percentages, while continuous variables were assessed for normality and expressed as mean ± standard deviation (SD) for normally distributed data or median (range) for non-normal distributions. Group comparisons between patients with and without PH were performed using the independent t-test for continuous variables and Fisher’s exact test for categorical variables.

To identify risk factors for PH, generalized linear models (GLM) with a log link and Poisson distribution with robust standard errors were used. This approach directly estimates relative risks (RRs), which are preferable to odds ratios in outcomes with high prevalence, and is robust when predictors may lie along an intermediate pathophysiologic continuum—such as structural cardiac changes related to ESRD and/or PH—thereby reducing the risk of over-adjustment. Both univariable and multivariable GLM analyses were performed, and results are presented as adjusted risk ratios (ARRs) with 95% confidence intervals (CIs). Statistical significance was defined as *p* < 0.05.

Linearity on the log-risk scale was assessed using LOWESS-smoothed plots for all continuous predictors. No meaningful deviations from linearity were observed; therefore, continuous variables were retained in their original untransformed form. Model fit was evaluated, and dispersion was considered acceptable for the Poisson generalized linear model.

## Results

### Patient selection and baseline characteristics

**Figure 1.**
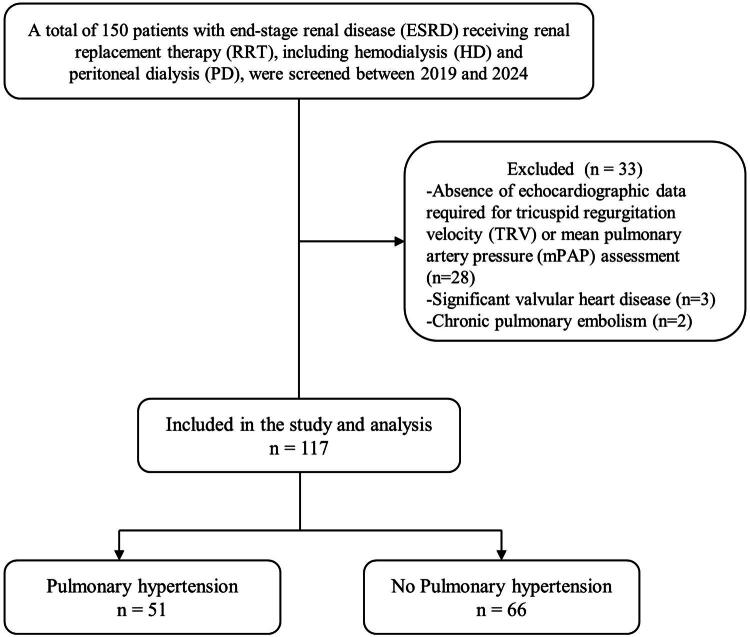
Flow diagram of patient selection. A total of 150 patients with end-stage renal disease receiving renal replacement therapy, including hemodialysis and peritoneal dialysis, were screened between 2019 and 2024. Twenty-eight patients were excluded due to absence of echocardiographic data required for TRV or mPAP assessment, three due to significant valvular heart disease, and two due to chronic pulmonary embolism. Consequently, 117 patients were included in the final analysis. Abbreviations: ESRD, end-stage renal disease; RRT, renal replacement therapy; HD, hemodialysis; PD, peritoneal dialysis; TRV, tricuspid regurgitation velocity; mPAP, mean pulmonary artery pressure.

Data from 150 patients with ESRD who received renal replacement therapy, including HD and PD, at our center between 2019 and 2024 were retrieved from the database (Figure 1). Twenty-eight patients were excluded due to the absence of echocardiographic data necessary for assessing TRV or mean PAP. An additional three patients were excluded due to significant valvular heart disease, and two were excluded due to chronic pulmonary embolism. Consequently, data from 117 patients were included in the final analysis. The mean age of the patients was 67.46 ± 15.67 years, and 49 (41.88%) were men. The causes of CKD included diabetic nephropathy (14.75%), hypertensive nephropathy (8.09%), lupus nephritis (1.84%), polycystic kidney disease (2.84%), and others (4.11%). The prevalence of PH was 43.6%. The demographic and clinical characteristics are summarized in Table 1.

**Table 1.** Baseline demographic and clinical characteristics of patients with end-stage renal disease according to the presence of pulmonary hypertension.

Characteristics	Total (*n* = 117)	PH (*n* = 51)	No PH (*n* = 66)	*P* value*
Age (years), mean ± SD	67.46 ± 15.67	66.88 ± 17.49	67.94 ± 14.53	0.722
Male, *n* (%)	49	19 (38.78)	30 (61.22)	0.451
BMI (kg/m^2^), mean ± SD	23.65 ± 7.34	22.72 ± 4.60	24.34 ± 8.87	0.232
Etiology of ESRD, *n* (%)				
Diabetes mellitus	60	28 (46.67)	32 (53.33)	0.577
Hypertension	78	34 (43.59)	44 (56.41)	1.000
Glomerulonephritis	1	1 (100.0)	0 (0)	0.436
Systemic lupus erythematosus	3	1 (33.33)	2 (66.67)	1.000
Unknown	25	10 (40.0)	15 (60.0)	0.821
Hypertension, *n* (%)	64	28 (43.75)	36 (56.25)	0.969
Diabetes mellitus, *n* (%)	111	47 (42.34)	64 (57.66)	0.401
Coronary artery disease, *n* (%)	29	16 (55.17)	13 (44.83)	0.195
Cerebrovascular accident, *n* (%)	11	4 (36.36)	7 (63.64)	0.754
Atrial fibrillation, *n* (%)	10	7 (70.0)	3 (30.0)	0.101
Heart Failure, *n* (%)	27	13 (48.15)	14 (51.85)	0.660
Type of RRT, *n* (%)				
Hemodialysis	86	40 (46.51)	46 (53.49)	0.398
Peritoneal dialysis	31	11 (35.48)	20 (64.52)	0.398
Dialysis vintage (years), mean ± SD median, IQR	1.52 ± 4.74 1 (0.67)	2.08 ± 6.99 1 (0.58)	1.06 ± 0.85 1 (0.75)	0.347
Arteriovenous fistula, % (AVF)	11	5 (45.45)	6 (54.55)	1.000
Current medication, *n* (%)				
Beta- blocker	49	25 (51.02)	24 (48.98)	0.190
Calcium channel blocker	70	27 (38.57)	43 (61.43)	0.190
ACEI inhibitor	6	2 (33.33)	4 (66.67)	0.695
ARB	44	20 (45.45)	24 (54.55)	0.848
Diuretics	59	29 (49.15)	30 (50.85)	0.265
Hydralazine	25	11 (44.0)	14 (56.0)	1.000
Erythropoietin	62	22 (35.48)	40 (64.52)	0.065

Abbreviations: BMI, body mass index; ESRD, end-stage renal disease; PH, pulmonary hypertension; RRT, renal replacement therapy; ACE, angiotensin-converting enzyme; ARB, angiotensin receptor blocker; AVF, arteriovenous fistula; SD, standard deviation.

*Note: Continuous variables were compared using the independent t-test. Categorical variables were compared using the Chi-square test or Fisher’s exact test, as appropriate, depending on expected cell counts.

### Laboratory findings

The laboratory data obtained from routine monitoring of patients with CKD for medication adjustment are shown in Table 2. Blood samples in the HD group were collected midweek, representing the longest interdialytic interval, while in the CAPD group samples were obtained in the morning before starting treatment on a convenient day. However, no significant differences were observed between patients with and without PH.

**Table 2.** Laboratory parameters of patients with end-stage renal disease according to the presence of pulmonary hypertension.

Parameters	Mean ± SD			*P* value
	Total	PH	No PH	
BUN, (mg/dL)	51.55 ± 32.40	48.69 ± 29.76	53.76 ± 34.36	0.404
Creatinine, (mg/dL)	6.58 ± 3.28	6.11 ± 3.11	6.95 ± 3.39	0.172
Hemoglobin (g/L)	10.24 ± 3.48	10.24 ± 3.79	10.34 ± 3.25	0.871
Calcium (mg/dL)	8.90 ± 0.88	8.95 ± 1.04	8.86 ± 0.75	0.610
Phosphorus (mg/dL)	4.14 ± 1.78	4.17 ± 1.75	4.12 ± 2.14	0.896
Calcium-phosphate product (mg^2^/dL^2^)	36.48 ± 18.67	36.83 ± 17.08	36.22 ± 19.89	0.864
Albumin (g/L)	3.31 ± 0.80	3.38 ± 0.91	3.25 ± 0.74	0.435
PTH (pg/mL)	362.45 ± 410.19	413.75 ± 497.16	328.90 ± 342.92	0.351

Abbreviations: BUN, blood urea nitrogen; PH, pulmonary hypertension; PTH, parathyroid hormone; SD, standard deviation; mg, milligram; dL, deciliter; g, gram; mL, milliliter; pg, picogram.

### Echocardiographic findings

Transthoracic echocardiography was performed in nearly all patients included in this study. The findings are summarized in Table 3. Patients with PH demonstrated significantly larger left ventricular diastolic dimension (45.80 ± 6.53 vs. 41.92 ± 7.26 mm, *p* = 0.003) and higher LV mass index (138.17 ± 30.83 vs. 122.68 ± 42.71 g/m^2^, *p* = 0.031) compared with those without PH. Although left ventricular ejection fraction tended to be lower in the PH group, the difference was not statistically significant (59.23 ± 13.81 vs. 62.95 ± 11.08%, *p* = 0.109).

**Table 3.** Echocardiographic findings of patients with and without pulmonary hypertension.

Parameters	Mean ± SD			*P* value*
	Total	PH	No PH	
Left ventricular diastolic dimension, mm	43.61 ± 7.18	45.80 ± 6.53	41.92 ± 7.26	0.003
LV mass index	129.43 ± 38.62	138.17 ± 30.83	122.68 ± 42.71	0.031
LVEF, %	61.31 ± 12.44	59.23 ± 13.81	62.95 ± 11.08	0.109
Diastolic dysfunction, *n* (%)				<0.001
Grade I	57 (100.0)	7 (12.28)	50 (87.72)	
Grade II	43 (100.0)	35 (81.40)	8 (18.60)	
Left atrial volume index, mL/m^2^	48.95 ± 45.65	59.32 ± 15.38	40.96 ± 14.29	<0.001
Normal RV function, *n* (%)	111 (100.0)	46 (41.44)	65 (58.56)	0.077
TRV max, m/sec	2.93 ± 0.62	3.43 ± 0.36	2.37 ± 0.29	<0.001
Mean PAP by Abbas’s formula	21.90 ± 9.47	29.94 ± 7.80	15.15 ± 3.68	<0.001
RAP	5.20 ± 3.69	6.47 ± 4.38	4.21 ± 2.70	0.001
Mitral regurgitation				<0.001
No	77	22 (28.57)	55 (71.43)	
Yes	40	29 (72.50)	11 (27.50)	
Pericardial effusion				0.127
No	89	35 (39.33)	54 (60.67)	
Yes	28	16 (57.14)	12 (42.86)	

Abbreviations: LV, left ventricle; LVEF, left ventricular ejection fraction; RV, right ventricle; TRV, tricuspid regurgitation velocity; PAP, pulmonary artery pressure; RAP, right atrial pressure; PH, pulmonary hypertension; mL, milliliter; sec, second; Abbas’s formula, echocardiographic method for estimating mean pulmonary artery pressure from TRV.

*Note: Continuous variables were compared using the independent t-test. Categorical variables were compared using the Chi-square test or Fisher’s exact test, as appropriate, depending on expected cell counts.

Diastolic dysfunction was markedly more prevalent among patients with PH. Most patients with PH exhibited Grade II diastolic dysfunction (81.4%), while Grade I dysfunction predominated in those without PH (*p* < 0.001). Similarly, left atrial volume index was significantly greater in the PH group (59.32 ± 15.38 vs. 40.96 ± 14.29 mL/m^2^, *p* < 0.001).

Right-sided parameters also differed significantly. The maximum TRV was higher in patients with PH (3.43 ± 0.36 vs. 2.37 ± 0.29 m/s, *p* < 0.001), with a corresponding increase in mean pulmonary artery pressure estimated by Abbas’ formula (29.94 ± 7.80 vs. 15.15 ± 3.68 mmHg, *p* < 0.001). Right atrial pressure was also elevated in the PH group (6.47 ± 4.38 vs. 4.21 ± 2.70 mmHg, *p* = 0.001).

Mitral regurgitation was significantly more frequent in patients with PH (72.5% vs. 27.5%, *p* < 0.001). In contrast, there was no significant difference in right ventricular function (*p* = 0.077) or pericardial effusion (*p* = 0.127) between groups.

### Risk factors for pulmonary hypertension

Multivariable regression analysis was performed to identify independent predictors of PH in patients with ESRD (Table 4). Candidate variables were selected from two domains: (1) echocardiographic findings with significant mean differences in the unpaired t-test (left atrial volume index, left ventricular diastolic dimension, and mitral regurgitation), and (2) clinically relevant variables that could influence pulmonary pressure, such as AF and pericardial effusion. The GLM with a log link were applied to estimate RR in both univariable and multivariable analyses.

**Table 4.** Multivariable regression analysis of factors associated with pulmonary hypertension in patients with end-stage renal disease.

Candidate variable			Uni-variable analysis		Multivariable analysis	
	PH	No PH	Crude RR (95% CI)	*P* value	Adjusted RR (95% CI)	*P* value*
Atrial fibrillation (*n*)	7	3	1.70 (1.07–2.71)	0.025	N/A	–
Dialysis vintage (years), (*n* = 66)	2.08 ± 6.99	1.06 ± 0.85	1.23 (1.10–1.38)	<0.001	1.50 (1.31–2.47)	0.009
Hemoglobin (g/L), (*n* = 116)	10.24 ± 3.79	10.34 ± 3.25	0.99 (0.93–1.07)	0.860	1.02 (0.89–1.18)	0.747
Left ventricular diastolic dimension (mm), (*n* = 117)	45.80 ± 6.53	41.92 ± 7.26	1.04 (1.01–1.06)	0.003	1.06 (1.01–1.12)	0.036
Left atrial volume index (mL/m^2^), (*n* = 108)	59.32 ± 15.38	40.96 ± 14.29	1.03 (1.02–1.04)	<0.001	1.01 (0.99–1.03)	0.315
Mitral regurgitation, (*n* = 117)	29 (72.50)	11 (27.50)	2.54 (1.70–3.79)	<0.001	2.00 (1.12–3.58)	0.020
Pericardial effusion, (*n* = 117)	16 (57.14)	12 (42.86)	1.45 (0.96–2.19)	0.075	0.94 (0.49–1.83)	0.857

Abbreviations: PH: pulmonary hypertension; ESRD: end-stage renal disease; RR: relative risk; CI: confidence interval; mm: millimeter; m: meter; mL: milliliter; SD: standard deviation.

*Note: Continuous variables were compared using the independent t-test. Categorical variables were compared using the Chi-square test or Fisher’s exact test, as appropriate, depending on expected cell counts.

**Figure 2.**
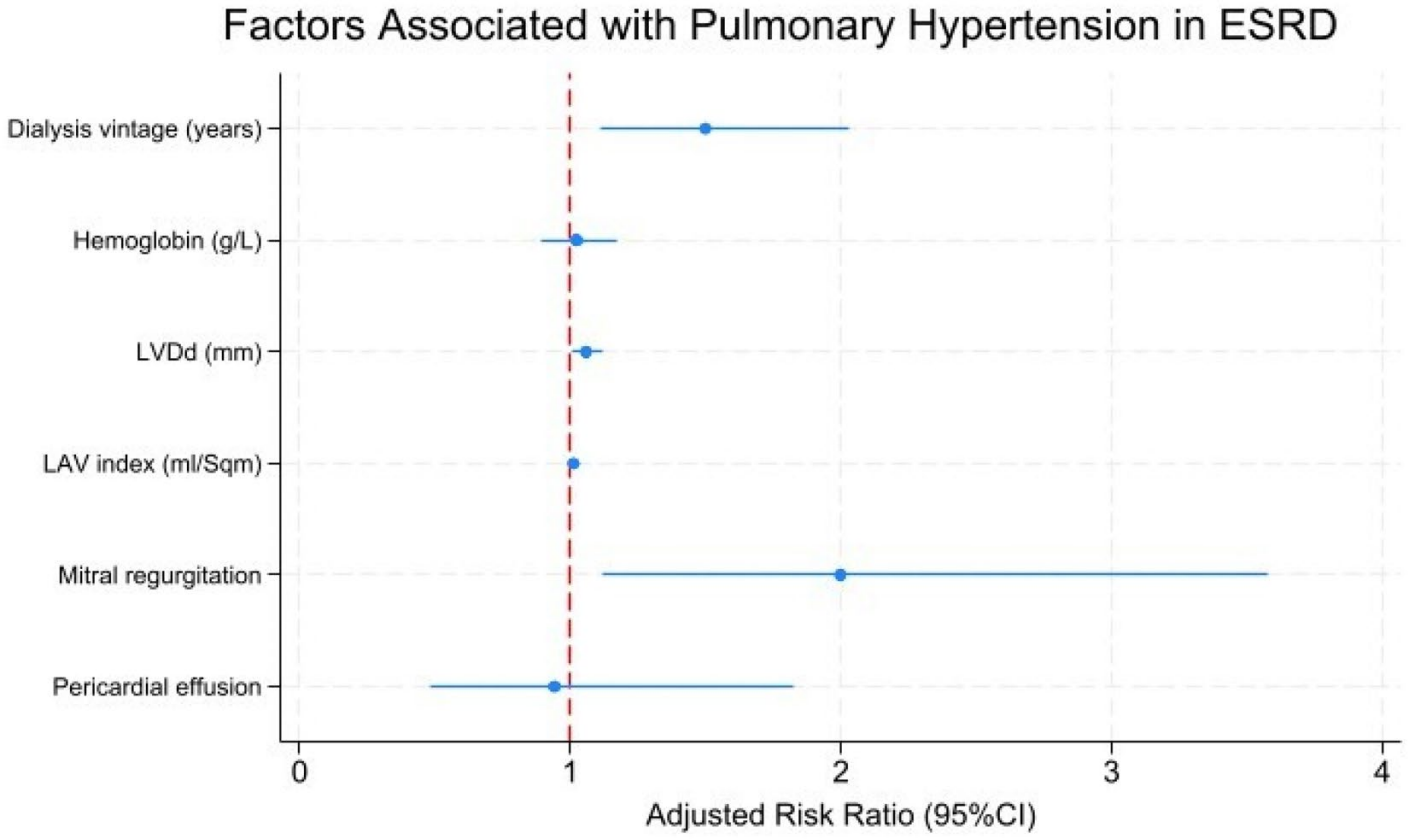
Adjusted relative risks for factors associated with pulmonary hypertension in ESRD (forest plot). Forest plot showing adjusted relative risks (RR) with 95% confidence intervals from a multivariable generalized linear model (GLM; log link, Poisson with robust standard errors). The model simultaneously included seven prespecified variables: AF (*n* = 117), dialysis vintage (years; *n* = 66), hemoglobin (g/L; *n* = 116), left ventricular diastolic dimension (mm; *n* = 117), left atrial volume index (mL/m^2^; *n* = 108), mitral regurgitation (*n* = 117), and pericardial effusion (*n* = 117). Squares represent adjusted RR; horizontal lines indicate 95% CI; the vertical reference line denotes RR = 1 (no association). Sample sizes reflect available data for each variable. Abbreviations: PH: pulmonary hypertension; ESRD: end-stage renal disease; RR: relative risk; CI: confidence interval; AF: atrial fibrillation; LV: left ventricle; LVDd: left ventricular diastolic dimension; LAVI: left atrial volume index; MR: mitral regurgitation; mm: millimeter; mL: milliliter.

In the univariable analysis, AF, longer dialysis vintage, larger left ventricular diastolic dimension, increased left atrial volume index, mitral regurgitation, and pericardial effusion were significantly associated with PH. After adjustment for confounders, only dialysis vintage (adjusted RR 1.50 per year, 95% CI 1.31–2.47, *p* = 0.009), left ventricular diastolic dimension (adjusted RR 1.06 per mmHg, 95% CI 1.01–1.12, *p* = 0.036), and mitral regurgitation (adjusted RR 2.00 yes/no variable, 95% CI 1.12–3.58, *p* = 0.020) remained independent predictors. Notably, AF showed a significant association in the univariable analysis (*p* = 0.025) but was omitted from the multivariable model due to collinearity, and thus no adjusted RR was reported. In summary, dialysis vintage, left ventricular diastolic dimension, and mitral regurgitation were identified as independent risk factors for PH. Details are provided in Table 4, and the multivariable analysis is summarized as a forest plot in Figure 2 for direct comparison across variables.

## Discussion

### Prevalence

In this study, the prevalence of PH was 43.6% (51 of 117 patients), consistent with prior echocardiography-based reports in ESRD/HD patients, which have ranged widely from 17% to over 70% [1,2,18]. Earlier criteria defined as PASP >35 mmHg estimated from TRV combined with RAP assessment, were prone to overestimation, particularly when echocardiography was performed before dialysis in volume-overloaded patients. TR jet velocity and RVSP often decrease substantially after HD [1], indicating that elevated values may reflect transient fluid retention rather than true PH. To minimize this bias, we applied TRV-based criteria combined with structural and functional evidence. Although we anticipated that this stricter definition would reduce prevalence, persistent abnormalities—including left atrial enlargement, diastolic dysfunction, and mitral regurgitation—remained common. These findings underscore that PH in ESRD is driven by multifactorial mechanisms rather than volume overload alone.

Regarding AF, previously reported in 10–33% of PH patients and 7–27% of ESRD/dialysis patients [19,20], we observed it in 10 of 117 patients (∼8.5%), which falls within these ranges. Importantly, our cohort maintained hemoglobin >10 g/dL and albumin >3 g/dL in both PH and non-PH groups, reflecting adequate treatment and indicating that anemia and malnutrition—major confounders—were unlikely to bias the results.

### AVF in HD not the principal cause of PH

When comparing dialysis modalities, PH was numerically more frequent in HD patients than in CAPD (46.5 vs. 35.5%), but this difference did not reach statistical significance. Thus, our results did not indicate AVF as the principal cause of PH in ESRD. While some case reports and small series have described improvement in pulmonary pressures following AVF revision or closure in selected patients [21,22], larger multicenter observational studies have failed to confirm a consistent effect of AVF flow on PH [23]. Moreover, long-term cohort data demonstrated that secondary PH after AVF creation was associated with higher mortality risk but did not increase AVF failure rates [24]. Taken together, these findings suggest that AVF may contribute in certain circumstances but is unlikely to be the dominant mechanism of PH in ESRD. Instead, other processes such as left-heart disease, long-term dialysis exposure, and systemic vascular dysfunction appear to play more central roles.

### Factors associated with PH and the role of cardiac structure

The GLM with a log link was applied instead of conventional logistic regression, given that the development of PH in ESRD involves a complex causal cycle with multiple intermediate outcomes (e.g. CKD → anemia/hypoalbuminemia → cardiac remodeling → PH). Logistic regression that treats PH as a simple binary outcome may oversimplify such relationships, whereas GLM provides more clinically interpretable estimates in the form of risk ratios and better accommodates the underlying pathophysiology [25].

Multivariable analysis identified three independent predictors of PH: dialysis vintage, left ventricular diastolic dimension, and mitral regurgitation. These can be grouped into three principal mechanistic domains:

*Structural cardiac remodeling from left-heart disease—*LV and LA abnormalities, including MR, support a post-capillary mechanism of PH driven by elevated filling pressures and diastolic dysfunction. Notably, LAVI showed significant differences in univariable analysis but was not retained in multivariable modeling, suggesting that its effect was mediated by related parameters such as LV dimension and MR. Thus, LAVI appears to represent an intermediate outcome (marker of diastolic burden) rather than an independent determinant of PH [26]. Because the causal contribution of mitral regurgitation to PH is well recognized only in cases of moderate-to-severe structural MR, we pre-specified the exclusion of patients with significant MR to avoid confounding and to ensure that PH identified in this study reflected ESRD-related pathophysiology rather than primary valvular disease. Mild MR, which remained in the cohort, is common in ESRD and typically represents functional regurgitation driven by chronic volume overload, anemia-induced high-output states, left atrial dilation, and diastolic dysfunction. These same hemodynamic disturbances also promote post-capillary PH. Therefore, the independent association observed between mild MR and PH in our multivariable model likely reflects a shared cardiorenal pathophysiological milieu rather than a direct causal effect of MR on pulmonary pressures.

AF was observed within the expected prevalence range for ESRD and PH populations. Clinically, AF in this context is more likely to reflect the cumulative effects of left-sided cardiac remodeling, elevated filling pressures, and diastolic dysfunction rather than acting as a primary determinant of PH. AF therefore appears to represent a downstream manifestation of the same structural and hemodynamic processes that contribute to post-capillary PH in ESRD.

*Dialysis vintage—*Longer dialysis duration was associated with greater risk of PH, likely reflecting the cumulative effects of chronic volume load, repeated hemodynamic stress, and CKD-related myocardial and vascular remodeling.

*Biochemical/metabolic milieu of CKD—*Although hemoglobin and albumin were not significantly different between PH and non-PH groups, both remain clinically important complications of CKD that can influence cardiopulmonary function [27]. In this cohort, their effects were attenuated by treatment (erythropoietin therapy, blood transfusions) and by improved nutrition following renal replacement therapy initiation. As a result, hemoglobin and albumin levels were adequately controlled, with mean values >10 and >3 g/dL, respectively.

**Figure 3.**
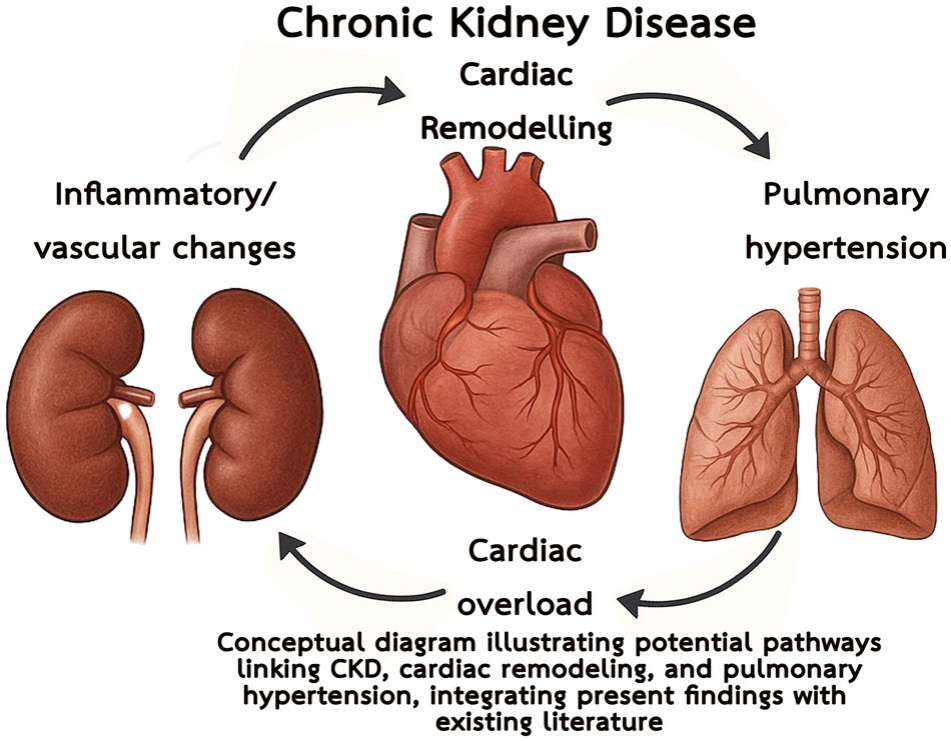
Conceptual pathways linking chronic kidney disease, cardiac remodeling, and pulmonary hypertension. The diagram illustrates a cyclical model in which CKD contributes to systemic inflammation and vascular changes, leading to structural cardiac remodeling and progressive cardiac overload. These changes predispose to post-capillary PH, which in turn aggravates cardiac dysfunction and venous congestion, further impairing renal function. Findings from this study—including the associations of dialysis vintage, left ventricular diastolic dimension, and mitral regurgitation with PH—support this multifactorial cycle and highlight PH in ESRD as part of a bidirectional heart–kidney–lung axis rather than a unidirectional outcome. Abbreviations: CKD, chronic kidney disease; PH, pulmonary hypertension; ESRD, end-stage renal disease; LV, left ventricle.

These findings can be integrated with current literature to postulate a mechanistic cycle linking CKD, cardiac remodeling, and PH (Figure 3) [28,29]. CKD-related inflammatory and vascular changes promote structural cardiac remodeling and overload [30], which in turn drive post-capillary PH. Elevated pulmonary pressures further exacerbate cardiac dysfunction and venous congestion, feeding back to worsen renal impairment [31]. Our results—particularly the associations of LV diastolic dimension, MR, and dialysis vintage with PH—are consistent with this cyclical model, suggesting that PH in ESRD should be conceptualized as part of a bidirectional heart–kidney–lung axis rather than a unidirectional outcome.

Taken together, these findings indicate that PH in this cohort is more likely to reflect post-capillary mechanisms related to left-heart disease, although definitive hemodynamic classification was not available. Future prospective longitudinal evaluation may help clarify whether PH in early dialysis vintage represents a potentially reversible condition or an early manifestation of progressive cardiopulmonary remodeling.

### Clinical implications

This study helps differentiate transient volume-related pulmonary hypertension from structural post-capillary pulmonary hypertension and underscores the clinical relevance of dialysis exposure duration and left ventricular remodeling in patient assessment.

### Limitations

This study has several limitations. First, echocardiography was performed in routine clinical practice without standardized timing relative to dialysis sessions, and interdialytic volume fluctuation may still contribute residual confounding despite use of TRV-based criteria. Second, pulmonary hypertension was defined echocardiographically without confirmation by right-heart catheterization. Third, the retrospective cross-sectional design precludes causal inference and cannot fully exclude reverse causality between pulmonary hypertension and left-sided cardiac remodeling, including mitral regurgitation and ventricular dilatation. Finally, the relatively small number of CAPD patients limited statistical power to assess modality-specific effects.

In Conclusion, PH in ESRD is not attributable to a single factor but rather reflects the combined impact of cardiac structural remodeling, cumulative dialysis exposure, and the biochemical milieu characteristic of CKD. This study represents the first systematic application of the full 2022 ESC/ERS TRV-based echocardiographic probability algorithm in a dialysis-only population, demonstrating that 43.6% of patients met criteria for PH, predominantly of post-capillary origin.

Future studies incorporating standardized dry-weight echocardiography, prospective designs with right-heart catheterization validation, and longitudinal outcome assessment are warranted to further refine diagnostic accuracy and clinical risk stratification in this population.

## Data Availability

The data that support the findings of this study are available from the corresponding author upon reasonable request. Due to patient privacy and ethical restrictions involving clinical data, the dataset cannot be made openly accessible.

## Abbreviations

PH: Pulmonary hypertension
ESRD: End-stage renal disease
TRV: Tricuspid regurgitation velocity
HD: Hemodialysis
CAPD: Continuous ambulatory peritoneal dialysis
AF: Atrial fibrillation

## References

[1] Yigla M, Nakhoul F, Sabag A, et al. Pulmonary hypertension in patients with end-stage renal disease. Chest. 2003;123(5):1577–1582. doi: 10.1378/chest.123.5.1577. 12740276

[2] Ramasubbu K, Deswal A, Herdejurgen C, et al. A prospective echocardiographic evaluation of pulmonary hypertension in chronic hemodialysis patients in the United States: prevalence and clinical significance. Int J Gen Med. 2010;3:279–286. doi: 10.2147/IJGM.S12946. 21042428 PMC2962323

[3] Pabst S, Hammerstingl C, Hundt F, et al. Pulmonary hypertension in patients with chronic kidney disease on dialysis and without dialysis: results of the PEPPER-study. PLoS One. 2012;7(4):e35310. doi: 10.1371/journal.pone.0035310. 22530005 PMC3329424

[4] Li Z, Liu S, Liang X, et al. Pulmonary hypertension as an independent predictor of cardiovascular mortality and events in hemodialysis patients. Int Urol Nephrol. 2014;46(1):141–149. doi: 10.1007/s11255-013-0486-z. 23793619

[5] Khomsanoi N, Chombandit T, Wiwatmanaskul J, et al. Economic evaluation of inpatient medication reconciliation with a subtraction strategy. Health Econ Rev. 2025;15(1):52. doi: 10.1186/s13561-025-00649-0. 40542934 PMC12181825

[6] Kreepala C, Ayutaya VSN, Phatthanakun R, et al. The biosensor using the target urine volatile organic compounds for detecting diabetic kidney disease. Sci Rep. 2025;15(1):14738. doi: 10.1038/s41598-025-00013-6. 40289139 PMC12034762

[7] Kreepala C, Srila-On A, Kitporntheranunt M, et al. The association between GFR evaluated by serum cystatin C and proteinuria during pregnancy. Kidney Int Rep. 2019;4(6):854–863. doi: 10.1016/j.ekir.2019.04.004. 31194092 PMC6551540

[8] Farber HW, Foreman AJ, Miller DP, et al. REVEAL Registry: correlation of right heart catheterization and echocardiography in patients with pulmonary arterial hypertension. Congest Heart Fail. 2011;17(2):56–64. doi: 10.1111/j.1751-7133.2010.00202.x. 21449993

[9] Zhang Q, Wang L, Zeng H, et al. Epidemiology and risk factors in CKD patients with pulmonary hypertension: a retrospective study. BMC Nephrol. 2018;19(1):70. doi: 10.1186/s12882-018-0866-9. 29554879 PMC5859392

[10] Li Y, Zhang Y, Wang J, et al. Pulmonary hypertension in end-stage renal disease patients on dialysis and pre-dialysis patients. Clin Invest Med. 2020;43(3):E44–E48. E8. doi: 10.25011/cim.v43i3.34631. 32971584

[11] He Y, Wang Y, Luo X, et al. Risk factors for pulmonary hypertension in maintenance hemodialysis patients: a cross-sectional study. Int Urol Nephrol. 2015;47(11):1889–1897. doi: 10.1007/s11255-015-1119-5. 26463080

[12] Kreepala C, Sangpanich A, Boonchoo P, et al. Measurement accuracy of total cell volume by automated dialyzer reprocessing: a prospective cohort study. Ann Med Surg (Lond). 2017;18:16–23. doi: 10.1016/j.amsu.2017.04.019. 28515906 PMC5425338

[13] Humbert M, Kovacs G, Hoeper MM, et al. 2022 ESC/ERS Guidelines for the diagnosis and treatment of pulmonary hypertension: developed by the task force for the diagnosis and treatment of pulmonary hypertension of the European Society of Cardiology (ESC) and the European Respiratory Society (ERS). Endorsed by the International Society for Heart and Lung Transplantation (ISHLT) and the European Reference Network on rare respiratory diseases (ERN-LUNG). Eur Heart J. 2022;43(38):3618–3731. doi: 10.1093/eurheartj/ehac237. 36017548

[14] Suthat Na Ayutaya V, Tantisatirapoon C, Aekgawong S, et al. Urinary cancer detection by the target urine volatile organic compounds biosensor platform. Sci Rep. 2024;14(1):3551. doi: 10.1038/s41598-024-54138-1. 38347076 PMC10861584

[15] Halloran PF, Madill-Thomsen KS, Reeve J. The molecular phenotype of kidney transplants: insights from the MMDx project. Transplantation. 2024;108(1):45–71. doi: 10.1097/TP.0000000000004624. 37310258 PMC10718223

[16] Yigla M, Fruchter O, Aharonson D, et al. Pulmonary hypertension is an independent predictor of mortality in hemodialysis patients. Kidney Int. 2009;75(9):969–975. doi: 10.1038/ki.2009.10. 19212417

[17] Abbas AE, Fortuin FD, Schiller NB, et al. Echocardiographic determination of mean pulmonary artery pressure. Am J Cardiol. 2003;92(11):1373–1376. doi: 10.1016/j.amjcard.2003.08.037. 14636929

[18] Bozbas S, Akcay S, Altin C, Bozbas H, Karacaglar E, Kanyilmaz S, et al. Pulmonary hypertension in patients with end-stage renal disease undergoing renal transplantation. In: Transplantation proceedings. Elsevier; 2009. doi: 10.1016/j.transproceed.2009.07.049. 19765426

[19] Genovesi S, Pogliani D, Faini A, et al. Prevalence of atrial fibrillation and associated factors in a population of long-term hemodialysis patients. Am J Kidney Dis. 2005;46(5):897–902. doi: 10.1053/j.ajkd.2005.07.044. 16253730

[20] Winkelmayer WC, Patrick AR, Liu J, et al. The increasing prevalence of atrial fibrillation among hemodialysis patients. J Am Soc Nephrol. 2011;22(2):349–357. doi: 10.1681/ASN.2010050459. 21233416 PMC3029907

[21] Raza F, Alkhouli M, Rogers F, et al. Case series of 5 patients with end-stage renal disease with reversible dyspnea, heart failure, and pulmonary hypertension related to arteriovenous dialysis access. Pulm Circ. 2015;5(2):398–406. doi: 10.1086/681266. 26064467 PMC4449253

[22] Amerling R, Malostovker I, Dubrow A, et al. Access High output heart failure in patients with upper arm A‐V fistulae: diagnosis and treatment. Hemodialysis International. 2005;9(1):70–71. doi: 10.1111/j.1492-7535.2005.1121a.x.

[23] Buryskova Salajova K, Malik J, Valerianova A. Pulmonary hypertension in patients on chronic hemodialysis. European Heart Journal. 2024;45(Supplement_1):ehae666. doi: 10.1093/eurheartj/ehae666.3261.

[24] Song L, Quan Z-L, Zhao L-Y, et al. Impact of pulmonary hypertension on arteriovenous fistula failure of hemodialysis patients: a 10 years follow-up cohort study. J Vasc Access. 2023;24(2):261–270. doi: 10.1177/11297298211027408. 34227421

[25] Zou G. A modified poisson regression approach to prospective studies with binary data. Am J Epidemiol. 2004;159(7):702–706. doi: 10.1093/aje/kwh090. 15033648

[26] Tsang TSM, Abhayaratna WP, Barnes ME, et al. Prediction of cardiovascular outcomes with left atrial size: is volume superior to area or diameter? J Am Coll Cardiol. 2006;47(5):1018–1023. doi: 10.1016/j.jacc.2005.08.077. 16516087

[27] Foley RN, Parfrey PS, Harnett JD, et al. Hypoalbuminemia, cardiac morbidity, and mortality in end-stage renal disease. J Am Soc Nephrol. 1996;7(5):728–736. doi: 10.1681/ASN.V75728. 8738808

[28] Agarwal R. Prevalence, determinants and prognosis of pulmonary hypertension among hemodialysis patients. Nephrol Dial Transplant. 2012;27(10):3908–3914. doi: 10.1093/ndt/gfr661. 22290987 PMC3484729

[29] Zeder K, Siew ED, Kovacs G, et al. Pulmonary hypertension and chronic kidney disease: prevalence, pathophysiology and outcomes. Nat Rev Nephrol. 2024;20(11):742–754. doi: 10.1038/s41581-024-00857-7. 38890546

[30] Glassock RJ, Pecoits-Filho R, Barberato SH. Left ventricular mass in chronic kidney disease and ESRD. Clin J Am Soc Nephrol. 2009;4 Suppl 1(Supplement_1):S79–S91. doi: 10.2215/CJN.04860709. 19996010

[31] Ronco C, McCullough P, Anker SD, et al. Cardio-renal syndromes: report from the consensus conference of the acute dialysis quality initiative. Eur Heart J. 2010;31(6):703–711. doi: 10.1093/eurheartj/ehp507. 20037146 PMC2838681

